# Huangqi Guizhi Wuwu decoction ameliorates myocardial damage in ovariectomized rats by regulating the structure and function of myocardial microvessels and intestinal flora

**DOI:** 10.1128/spectrum.00216-25

**Published:** 2025-08-04

**Authors:** Yanhua Jiang, Ying Yang, Jing Hu, Yanjun Liu, Haixia Liu, Zhiguo Zhang, Yanjing Chen

**Affiliations:** 1 Institute of Basic Theory for Chinese Medicine, China Academy of Chinese Medical Sciences 534975 , Beijing, China; 2 Yantai Hospital of Traditional Chinese Medicine 598841, Yantai, China; The Chinese University of Hong Kong, Hong Kong, China

**Keywords:** Huangqi Guizhi Wuwu decoction, ovariectomized rats, myocardial microvessels, intestinal flora, myocardial damage

## Abstract

Huangqi Guizhi Wuwu decoction (HGWD) is commonly used to treat cardiovascular diseases (CVDs), and HGWD could regulate intestinal flora and myocardial microvessels, this study aimed to examine whether HGWD could ameliorate myocardial damage in ovariectomized (OVX) rats by regulating the structure and function of myocardial microvessels and intestinal flora. A model of OVX rats was created, and histological examination through hematoxylin and eosin staining alongside TEM was employed to assess the microstructural lesions present in the myocardium. Myocardial microvasculature density was assessed using immunofluorescent staining. The systolic and diastolic properties of myocardial capillaries, coagulation function, blood rheology indicators, and inflammatory factor levels were evaluated. The 16S rRNA amplicon sequencing was used to detect intestinal flora in OVX rats and the intervention effect of HGWD. PICRUSt software was used to conduct a functional prediction analysis of the screened differential intestinal flora. HGWD ameliorated OVX-induced myocardium damage in rats. HGWD increased the density of myocardial microvessels and ameliorated their structural abnormalities. Additionally, HGWD maintained plasma endothelial nitric oxide synthase/endothelin-1 and prostacyclin/thromboxane balance, decreased plasma vascular endothelial-derived growth factor, thrombomodulin, endothelial protein C receptor, and von Willebrand factor levels, decreased the concentrations of serum inflammatory markers interleukin (IL)-1β and IL-6, reduced plasma viscosities, and improved the coagulation function. Besides, HGWD improved the structure and function of intestinal flora and altered significant alterations in the abundance of certain phylum and genus levels closely associated with CVD, including *Actinobacteria*, *Ruminococcus_1,* and *Roseburia*. HGWD alleviates myocardial damage in OVX rats, accompanied by structural remodeling of cardiac microvasculature and gut microbiota.

**IMPORTANCE:**

This work elucidated the impact of Huangqi Guizhi Wuwu decoction (HGWD) on cardiac microvasculature and gut microbiota. We observed that HGWD administration ameliorated ovariectomized-induced myocardium damage. HGWD not only increases myocardial microvascular density and repairs structural abnormalities but also balances vasoactive substances, reduces inflammatory factor levels, improves blood rheology, and coagulation function. Furthermore, it reshapes gut microbiota structure and function while modulating the abundance of microbial taxa closely associated with cardiovascular diseases (CVDs). The results highlight the unique advantages of traditional Chinese medicine in regulating systemic microenvironment homeostasis, suggesting that its multi-component, multi-pathway characteristics may offer more integrative intervention strategies for preventing and managing complex metabolic CVDs.

## INTRODUCTION

The incidence of cardiovascular diseases (CVDs) increases rapidly after menopause (1). Multiple pieces of evidence have highlighted the importance of estrogen in protecting women from CVDs (2). A previous study reported that estrogen levels could affect the structure and function of the intestinal flora (3). Increasing evidence from animal and human studies indicated that the composition and metabolic profiles of the intestinal flora are associated with the pathogenesis of CVDs (4). Furthermore, the structural and functional abnormalities of coronary microvasculature are linked to adverse clinical results in patients with various CVDs (5), and menopausal women are more susceptible to coronary microcirculation disorders (6). Estrogen deficiency-induced microcirculation disorders are implicated in postmenopausal CVDs.

Huangqi Guizhi Wuwu decoction (HGWD) is commonly used to treat CVDs and significantly protects the cardiovascular system (7). A previous study reported that HGWD improved hemorheology and inhibited inflammatory response after percutaneous coronary intervention for acute myocardial infarction (8). HGWD significantly improves the abnormal intestinal flora metabolism caused by paclitaxel, while Astragalus polysaccharide facilitates weight loss through intestinal flora regulation in the hyperlipid-induced obese mouse model (9). Endothelial cells are the innermost structure of the coronary microvessel, and their structural or functional abnormalities directly cause coronary microcirculation disorders. The ingredients in HGWD exert a protective effect on the vascular endothelium (10, 11). It is unknown whether HGWD ameliorates myocardial damage in ovariectomized (OVX) rats by improving myocardial microvessels and intestinal flora.

## MATERIALS AND METHODS

### Preparation of medicines

Composition and dosage of HGWD: *Astragalus membranaceus* (Fisch). (Astragalus) 9 g, *Cinnamomum cassia* Presl. (cassia twig) 9 g, *Paeonia suffruticosa* Andr. (peony) 9 g, *Ziziphus jujuba* Mill. (jujube) 9 g, and *Zingiber officinale* Rosc. (ginger) 18 g. The aforementioned herbs were immersed in water at a ratio of eight times their combined weight and subjected to refluxing on two occasions for 1 h each session. The two decoction liquids were mixed, concentrated into a crude drug at 1 g/mL, and stored at 4°C.

### Establishment of the OVX model and groups

Female Sprague-Dawley (SD) rats (*n* = 32, 200 ± 30 g, 10 weeks of age) were sourced from Beijing Vital River Laboratory Animal Technology Company Limited (certificate number: SCXK (Jing) 2016-0002). This study included a bilateral ovarian rat model. Prior to the excision of the ovaries, rats received abdominal cavity injections of 3% sodium pentobarbital anesthesia. The 24 rats that were effectively modeled were randomly assigned to three groups: OVX, E, and HGWD. The remaining eight rats were designated as the SHAM group, and small adipose tissue fragments beside the ovaries were removed without removing the ovaries. One week after the surgery, rats in both the SHAM and OVX groups were administered an equal volume of purified water, the E group rats received 0.18 mg/kg estrogen solution, and the HGWD group rats received 10 g/kg HGWD. These doses markedly enhanced the cardiac function in rats suffering from diabetic myocardial infarction (12). All rats received the drug once daily, and the treatment lasted 16 weeks.

### Pathological morphology and ultrastructure of myocardium

The myocardium was fixed using 4% paraformaldehyde solution, subsequently embedded in paraffin, sectioned to a thickness of 7 µm, and then subjected to staining with hematoxylin and eosin (HE). The left ventricle tissues (3 × 3 × 1 mm) were preserved using an electron microscope fixative. Subsequently, they were sliced into sections with a thickness of 50 nm and stained with uranyl acetate-lead citrate. The myocardial pathological changes and ultrastructure were examined using a light microscope (NIKON ECLIPSE C1 upright fluorescence microscope, Japan) and transmission electron microscopy (TEM; H7650, Hitachi Company, Japan), respectively.

### Immunofluorescent staining

The myocardium was fixed in 4% paraformaldehyde, paraffin-embedded and sectioned, and subjected to immunofluorescence staining. Cardiomyocyte nuclei were visualized using DAPI staining. Myocardial microvessels were identified by CD34 (Wuhan Seville Biotechnology Company Limited, GB13013) labeling and subsequently cultured at a temperature of 4°C. Following this, the samples were incubated with CY3-labeled goat anti-rabbit antibody (Wuhan Seville Biotechnology Company Limited, GB21303) to visualize the staining. The criteria for evaluating microvessels were as follows. All vascular endothelial cells or clusters of endothelial cells stained red were considered one blood vessel. Microvessels with lumens >8 red blood cells (RBCs) and thick muscular layers were not counted. Specific counting methods were as follows: The endocardial region with a higher density of microvessels was identified using a low-power microscope, and the microvessels in five high-power fields (200×) were counted to obtain average values. The microvessel density was quantified using the number of microvessels per field of view.

### Enzyme-linked immunosorbent assay (ELISA)

The levels of plasma or serum thrombomodulin (TM), endothelial protein C receptor (EPCR) (Wuhan Unisun Trading Company Limited, L200710857 and L200710855), endothelin-1 (ET-1), endothelial nitric oxide synthase (eNOS), thromboxane (TXA_2_), prostacyclin (PGI_2_), vascular endothelial-derived growth factor (VEGF), von Willebrand factor (vWF), interleukin (IL)-1β, and IL-6 (Nanjing Jiancheng Institute of Bioengineering, 20190904, 20191014, 20191012, 20190928, 20191014, 20190921, 20201120, and 20201205) were detected using ELISA.

### Detection of estrogen level, hemorheology, and coagulation function

E2 radioimmunoassay kit for estrogen, sourced from Beijing Northern Institute of Biotechnology (BFS20191101), was used to assess E2 level. Whole blood viscosity was evaluated using the cone and plate technique, while plasma viscosity was assessed through the capillary method. The coagulation method was used to detect APTT, PT, TT, and FIB levels (Beijing Seikexide Technology Development Company Limited, 2019-312208, 2019-314204, 2019-314205, 2019-313403).

### 16S rRNA amplicon sequencing

Fresh fecal samples were collected from all rats before being transferred to sterile cryopreserved tubes. The samples were promptly frozen at –80°C after collection. MagPure Soil DNA LQ Kit (Magen, Guangdong, China) was used to extract bacterial DNA from the feces. NanoDrop 2000 spectrophotometer (Thermo Fisher Scientific, Waltham, MA, USA) and agarose gel were used to verify the quality and quantity of DNA, respectively. The DNA served as a template for the amplification of bacterial 16S rRNA genes through RT-qPCR, utilizing barcoded primers in conjunction with Takara Ex Taq (Takara, Shiga, Japan). The PCR products were purified using Agencourt AMPure XP beads (Beckman Coulter Co., Brea, CA, USA) and quantified using a Qubit dsDNA Assay Kit (YEASEN, Shanghai, China). Sequencing was performed using Illumina NovaSeq 6000 (Illumina Inc., OE Biotech Company, Shanghai, China). Alpha diversity was estimated using the Shannon index, Simpson index, and Chao1 richness. Group comparisons were performed with the Kruskal-Wallis test (for >2 groups) or the Mann-Whitney *U*-test (for two groups), followed by Dunn’s post hoc test for multiple comparisons. Beta diversity was assessed via Bray-Curtis dissimilarity and tested using PERMANOVA with 999 permutations. All *P* values from taxonomic analyzes and correlation tests were adjusted for multiple testing using the Benjamini-Hochberg false discovery rate correction.

PICRUSt software with the protein orthologous clusters (COG), Kyoto Encyclopedia of Genes and Genomes (KEGG) was used to predict the composition of the gene function of microbes.

### Statistical analysis

Data are presented as $\bar{X}$ ± s. Statistical analyzes were conducted using IBM SPSS 25.0, and GraphPad Prism 9 was employed for the generation of statistical graphs. One-way ANOVA analysis was implemented to assess differences among multiple groups, the LSD test was applied under conditions of homogeneity of variances, and Kruskal-Wallis was utilized for nonparametric comparisons in cases of variance heterogeneity. For comparisons between pairs of groups, the Bonferroni method was applied. It was judged statistically significant if *P* ˂ 0.05.

## RESULTS

### Effect of HGWD on body weight and the level of E2 in OVX rats

**Fig 1:**
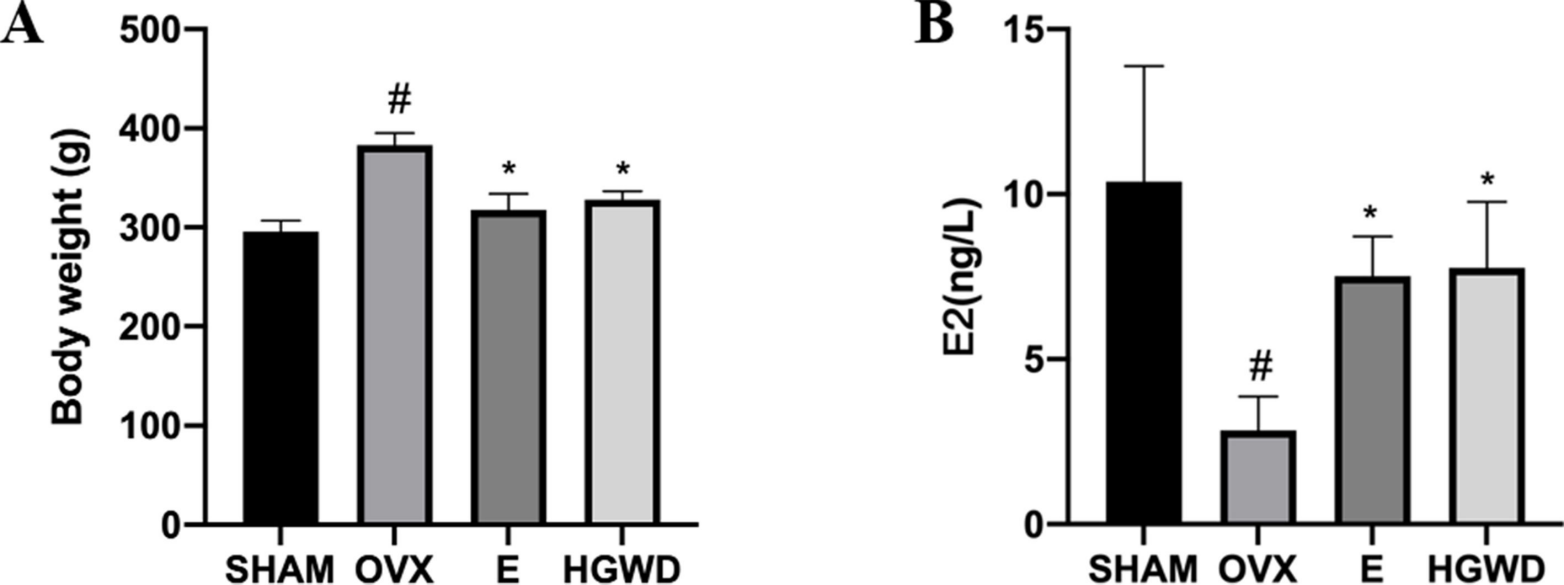
Effect of HGWD on body weight and the level of E2 in OVX rats. (**A**) Body weight (*n =* 8). (**B**) The level of E2 (*n =* 8). In comparison to the SHAM group, ^#^*P* < 0.05. In comparison to the OVX group, *P < 0.05.

The rats in the OVX group exhibited an increased body weight compared to those in the SHAM group, while their E2 levels were found to be reduced (Fig. 1A and B). The body weight in the E and HGWD groups was lower, and the E2 level of rats in the E and HGWD groups was higher than those of rats in the OVX group.

### Effect of HGWD on pathological changes of myocardial tissue in OVX rats

**Fig 2:**
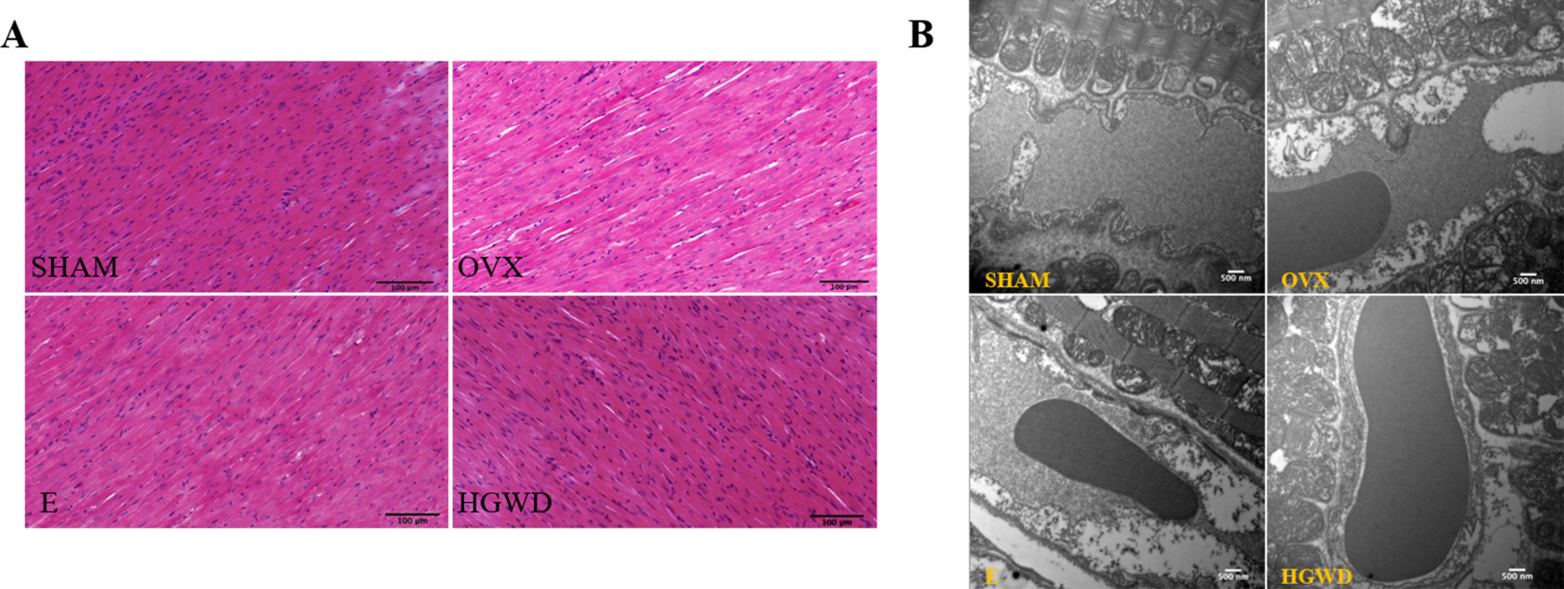
Effect of HGWD on pathological changes of myocardial tissue in OVX rats. (**A**) HE staining of the myocardium (200×, scale bar = 100 µm). (**B**) Myocardial ultrastructure (15,000×, scale bar = 500 nm).

HE staining demonstrated that the cardiomyocytes from rats in the OVX group exhibited greater atrophy and displayed more pronounced intercellular spaces compared to those from the SHAM group (Fig. 2A). The cardiomyocytes of rats in the E and HGWD groups exhibited occasional atrophy and focal stromal hyperplasia. TEM showed that some myocardial cells from rats in the OVX group showed pyknotic nuclei. Additionally, the mitochondrial cristae in these cells appeared to be less dense and more swollen in comparison to those observed in the SHAM group (Fig. 2B). Cardiomyocyte myofilaments of rats in the E and HGWD groups were more neatly arranged, with intact mitochondrial membranes, and their mitochondrial cristae were clearer without edema than those of rats in the OVX groups.

### Effects of HGWD on myocardial microvasculature and endothelial cells in OVX rats

**Fig 3:**
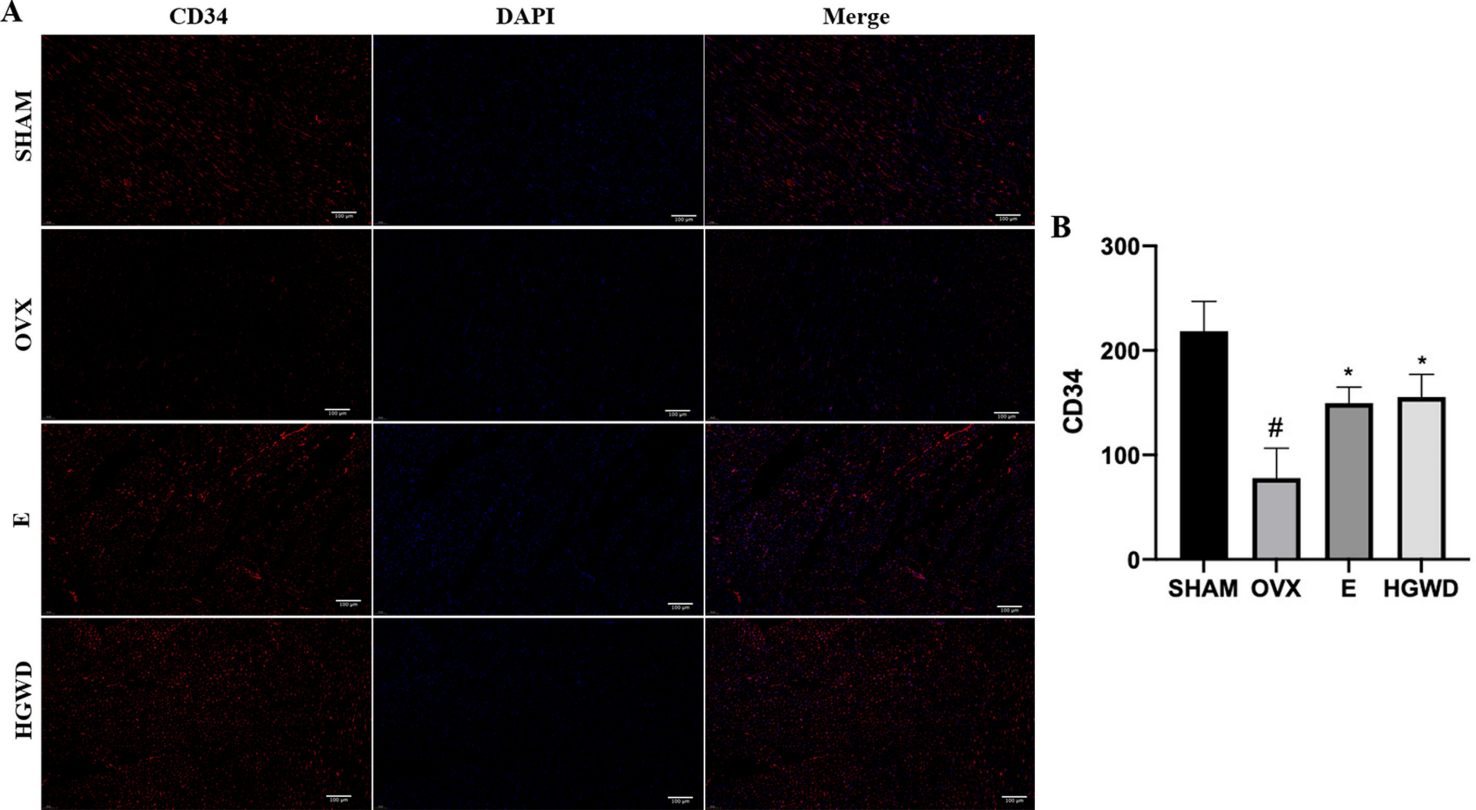
Effects of HGWD on myocardial microvasculature and endothelial cells in OVX rats. (**A**) CD34 immunofluorescence (200×, scale bar = 100 µm). CD34 is highly expressed in the endothelial cells of newly formed blood vessels, especially during angiogenesis. Therefore, CD34 is commonly used as a marker for newly formed blood vessels to assess microvascular density in tissues. (**B**) The expression of CD34 (*n =* 8). In comparison to the SHAM group, ^#^*P* < 0.05. In comparison to the OVX group, *P < 0.05.

To examine the microvascular density of the myocardium and observe the condition of endothelial cells, immunofluorescence and electron microscopy were utilized. DAPI blue fluorescence was used to stain the cardiomyocyte nuclei, representing cardiomyocytes, and CD34 red fluorescence marked the myocardial microvessels. The density of myocardial microvessels in the OVX rats was markedly reduced compared to that observed in the SHAM group (Fig. 3A and B). The density of myocardial microvessels in rats belonging to the E and HGWD groups was markedly elevated compared to that observed in the OVX group. In addition, the myocardial microvascular endothelial cells in the OVX group had obvious swelling (Fig. 2B) and cytoplasmic cavitation. The edema range of myocardial microvascular endothelial cells in the E and HGWD groups was limited.

### Effects of HGWD on the structural integrity of endothelial cells, coagulation function, and hemorheology in OVX rats

**Fig 4:**
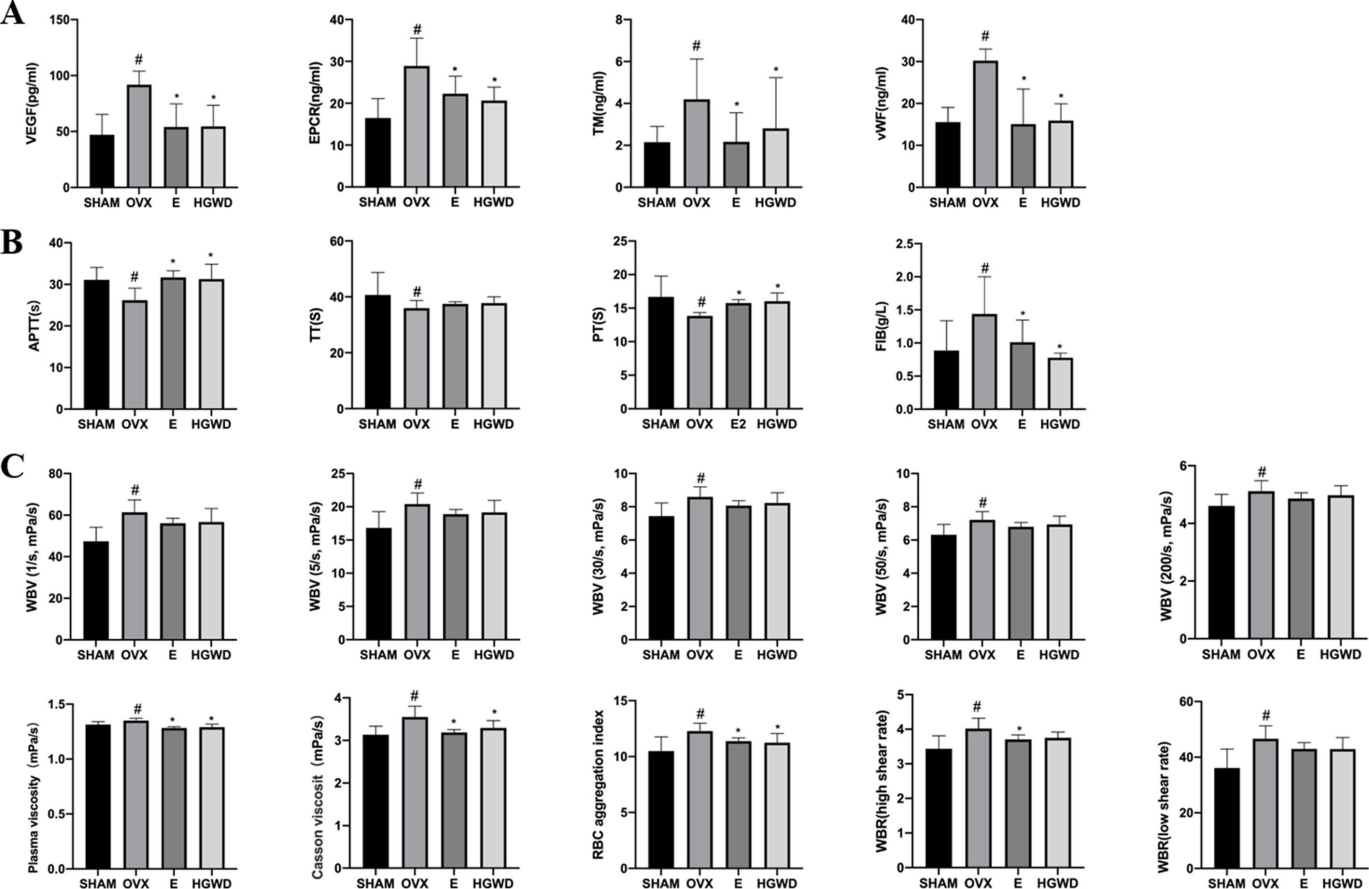
Effects of HGWD on the structural integrity of endothelial cells, coagulation function, and hemorheology in OVX rats. (**A**) Vascular endothelial function indicators (*n =* 8). (**B**) Coagulation function (*n =* 8). (**C**) Blood viscosity (*n =* 8). In comparison to the SHAM group, ^#^*P* < 0.05. In comparison to the OVX group, *P < 0.05.

VEGF is associated with endothelial growth and angiogenesis. EPCR and TM are sensitive and specific molecular markers for microvascular endothelial injury, commonly used to evaluate the structural integrity of endothelial cells. vWF is an active factor secreted by microvascular endothelial cells, which is an important biomarker for endothelial cell activation and damage. VEGF, EPCR, TM, and vWF levels were markedly elevated in the OVX group of rats compared to those in the SHAM group (Fig. 4A). VEGF, EPCR, TM, and vWF levels were significantly lower among rats in the HGWD and E groups than among those in the OVX group.

When endothelial cells are damaged, plasma vWF levels will significantly increase, mediating platelet adhesion and aggregation, and keeping the blood in a hypercoagulable state. The coagulation method was used to detect four coagulation parameters, and hemorheology was used to detect blood flow status. In contrast to the SHAM group, the OVX group exhibited reduced levels of APTT, TT, and PT, alongside elevated FIB levels. Conversely, both the HGWD and E groups demonstrated increased APTT, TT, and PT levels, coupled with diminished FIB levels (Fig. 4B). Rats in the OVX group exhibited higher whole blood viscosity under low-cut (1 and 30 s^–1^), mid-cut (50 s^–1^), high-cut (200 s^–1^), cassone, and plasma viscosities; the whole blood viscosity under low-cut (5 s^–1^), RBC aggregation index, whole blood high-cut relative index, and whole blood low-cut relative index was more increased than those of rats in the SHAM group (Fig. 4C). Rats in the E group exhibited lower cassone and plasma viscosities, the HGWD group exhibited lower cassone and plasma viscosities than those in the OVX group.

### Effects of HGWD on microvascular relaxation and contraction activity, inflammatory factors in OVX rats

**Fig 5:**
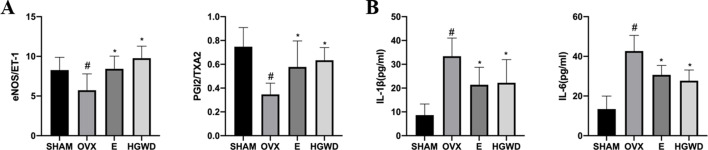
Effects of HGWD on microvascular relaxation and contraction activity, inflammatory factors in OVX rats. (**A**) Plasma systolic and diastolic factors (*n =* 8). (**B**) Serum inflammatory factors (*n =* 8). In comparison to the SHAM group, ^#^*P* < 0.05. In comparison to the OVX group, *P < 0.05.

eNOS, PGI_2_, ET-1, and TXA_2_ are active factors secreted by endothelial cells and are key regulatory factors for microvascular relaxation and contraction activity. The eNOS/ET-1 and PGI_2_/TXA_2_ ratios observed in the OVX group of rats were markedly lower compared to those in the SHAM group. In contrast, the eNOS/ET-1 and PGI_2_/TXA_2_ ratios in the HGWD and E groups of rats were significantly elevated relative to the OVX group (Fig. 5A).

The concentrations of inflammatory mediators, specifically IL-1β and IL-6, were markedly elevated in the rats belonging to the OVX group in comparison to the SHAM group. Conversely, the levels of IL-1β and IL-6 observed in the E and HGWD groups were significantly reduced relative to those in the OVX group (Fig. 5B).

### Effects of HGWD on comparison of intestinal flora in OVX rats

**Fig 6:**
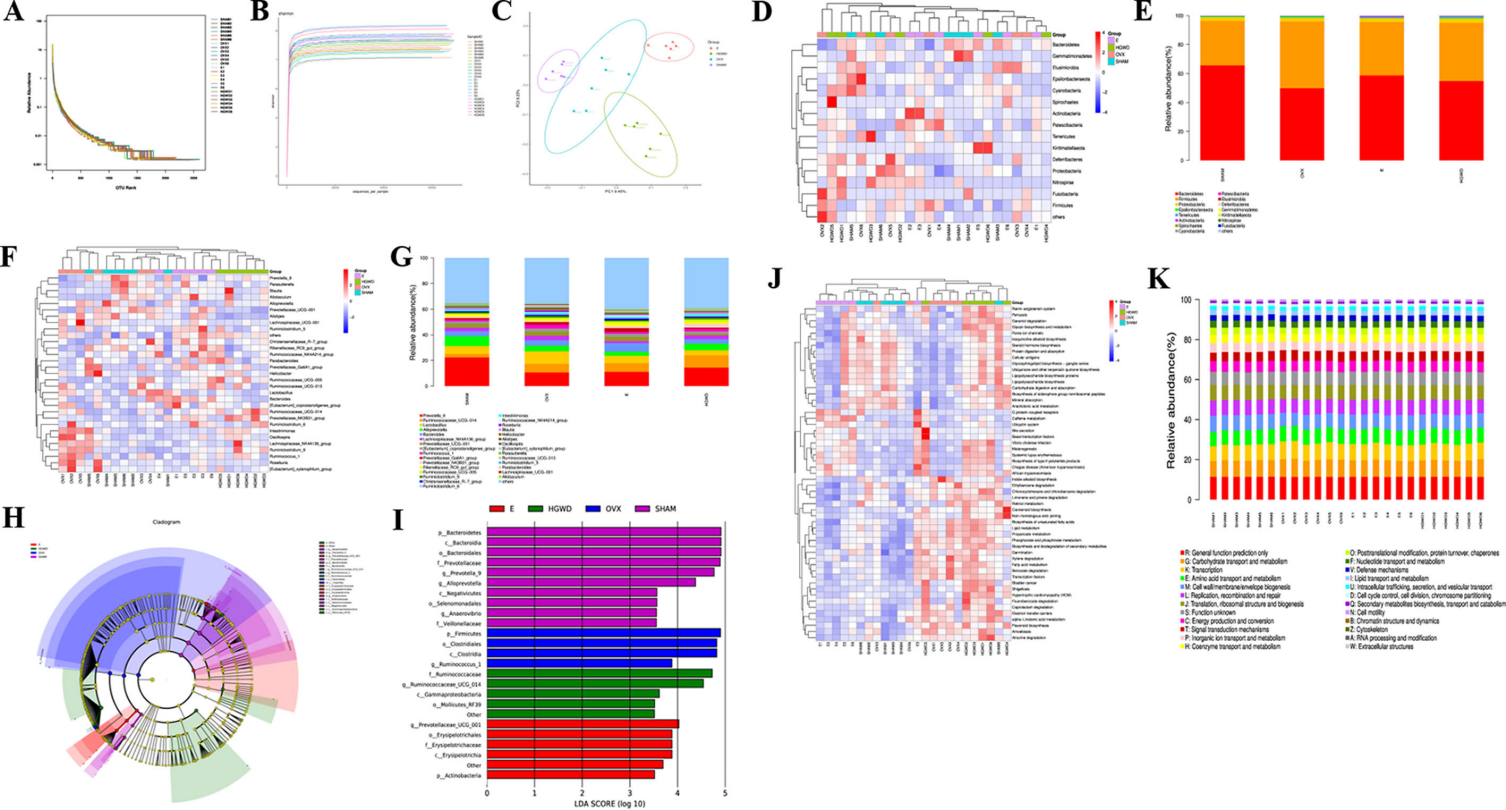
Effects of HGWD on comparison of intestinal flora and PICRUSt function prediction analysis in OVX rats. (**A and B**) Ran-Abundance curves, sparse curves, and Shannon index of α diversity. (**C**) PCoA of β diversity. (**D and E**) Taxonomic classification of the gut microbiome illustrating the proportion of microorganisms in each group at the phylum level. (**F and G**) Taxonomic classification of the gut microbiome, illustrating the proportion of microorganisms in each group at the genus level. (**H**) Cladogram of LefSe analysis. (**I**) LDA scores in each group. (**J**) Heatmap of KEGG terms. (**K**) COG barplot.

The rank-abundance curves (Fig. 6A) and sparse curves (Fig. 6B) were stabilized, indicating that the sequencing data is valid. UniFrac-based principal coordinate analysis (PCoA) revealed that the microbiota composition for each group exhibited distinct clustering and can be separated (Fig. 6C), indicating that HGWD could modulate the gut microbial imbalance in the OVX group. As shown in Fig. 6D and E, the histogram and heatmap at the phylum level illustrated that *Firmicutes* and *Bacteroidetes* were the predominant species in each group. Notably, rats belonging to the OVX group showed a significant reduction in *Firmicutes* and an elevation in *Bacteroidetes*, while those in the HGWD and E groups demonstrated an opposite trend. Additionally, *Actinobacteria* was higher among rats in the HGWD group than among those in the OVX group, while *Actinobacteria* and *Spirochaetes* were higher among rats in the E group than among those in the OVX group. At the genus level (Fig. 6F and G), in comparison to the SHAM group, the OVX group exhibited a reduction in the abundance of *Prevotella_9*, *Alloprevotella,* and *Parasutterella*, while an increase was observed in the levels of *Ruminococcus_1*, *Ruminococcaceae_UCG-014*, and *Roseburia*. In comparison to the OVX group, rats in the HGWD group exhibited a decreased abundance of *Prevotellaceae_UCG-001*, *Alistipes*, *Ruminococcus_1*, and *Roseburia*, whereas the prevalence of *Prevotellaceae_NK3B31_group* was found to be elevated. In the E group, *Rikenellaceae_RC9_gut_group* and *Ruminococcus_1* were decreased, and an increase was observed in the levels of *Lachnospiraceae_NK4A136_group*, *Ruminococcaceae_NK4A214_group*, and *Bacteroides*.

The LEfSe analysis was performed to search for biomarkers with statistical significance among the SHAM, OVX, HGWD, and E groups (Fig. 6H and I). A larger linear discriminant analysis (LDA) score indicates a stronger impact of species abundance on the observed differential effect. The analysis revealed that *f__Veillonellaceae*, *g__Anaerovibrio*, *o__Selenomonadales*, *c__Negativicutes*, *g__Alloprevotella*, *g__Prevotella_9*, *f__Prevotellaceae*, *o__Bacteroidales*, *c__Bacteroidia*, and *p__Bacteroidetes* exhibited larger LDA values among rats belonging to the SHAM group. These taxa may serve as potential biomarkers for characterizing the microbial composition within the SHAM group. The LDA values of the OVX group were in the order of *g__Ruminococcus_1*, *c__Clostridia*, *o__Clostridiales*, and *p__Firmicutes* and could be used as biomarkers in the OVX group. *o__Mollicutes_RF39*, *c__Gammaproteobacteria*, *g__Ruminococcaceae_UCG_014*, and *f__Ruminococcaceae* could be used as biomarkers among rats in the HGWD group, and *p__Actinobacteria*, *c__Erysipelotrichia*, *f__Erysipelotrichaceae*, *o__Erysipelotrichales*, and *g__Prevotellaceae_UCG_001* could be used as a biomarker among rats in the E group.

### Effects of HGWD on PICRUSt function prediction analysis in OVX rats

The heatmap of KEGG and COG bar plots (Fig. 6J and K) predicted that HGWD can regulate carbohydrate digestion and absorption, lipid and fatty acid metabolism, and lipopolysaccharide biosynthesis, indicating that HGWD primarily regulates glucose and lipid metabolism *in vivo* by improving the structure and function of intestinal flora in OVX rats.

## DISCUSSION

This study examined the protective effect of HGWD on the structure and function of myocardial microvasculature and intestinal flora in OVX rats. OVX caused weight gain and a significant decrease in plasma E2 levels. E and HGWD enhanced plasma E2 levels in OVX rats. Cardiomyocytes in OVX rats exhibited signs of atrophy, characterized by an increase in the intercellular spaces, some nuclei shrank, and a notable reduction and swelling of the mitochondrial cristae. HGWD ameliorated the atrophy and interstitial hyperplasia observed in the myocardial cells of OVX rats, preserving the structural integrity of mitochondrial membranes and cristae.

HGWD enhanced plasma E2 levels in OVX rats, suggesting that HGWD may have phytoestrogen activity. HGWD contains bioactive compounds that may exert estrogen-like effects and modulate aromatase activity. Isoflavone upregulates estrogen receptor β expression levels (13), which may act as selective estrogen receptor modulators to mimic E2 effects (14). HGWD contains bioactive compounds to upregulate aromatase CYP19A1 (15), potentially enhancing androgen-to-estrogen conversion in OVX rats.

A previous study reported that changes in myocardial microvascular structure or function are implicated in CVD pathogenesis (16). Simultaneously, estrogen regulates the structural and functional integrity of myocardial microvessels, particularly the secretory function of microvascular endothelial cells (17). This study demonstrated the changes in the myocardial microvessel density and structure of OVX rats, including decreased myocardial microvessel density and edema of microvascular endothelial cells. HGWD enhanced myocardial microvessel density, ameliorated the microvascular endothelial cell edema, and protected myocardial microvessels.

Studies have shown that endothelial secretion dysfunction causes eNOS and PGI_2_ levels reduction, ET-1 and TXA_2_ elevation, disrupting the balanced state of eNOS/ET-1 and PGI_2_/TXA_2_, thereby inducing microvascular contractile dysfunction (18). The findings revealed that plasma eNOS/ET-1 and PGI_2_/TXA_2_ ratios decreased in OVX rats, while estrogen and HGWD increased rat plasma eNOS/ET-1 and PGI_2_/TXA_2_ ratios. Hemorheological changes impaired microvascular blood flow (19), with increased blood viscosity adversely affecting microcirculatory blood flow (20). Additionally, vWF is an active factor secreted by microvascular endothelial cells that regulates blood viscosity and coagulation function. Herein, the plasma vWF of OVX rats increased, while HGWD lowered plasma vWF levels and potentially enhanced microcirculation blood flow. The coagulation function revealed that the APTT, TT, and PT of OVX rats were decreased, indicating a state of hypercoagulability in their blood. HGWD prolonged APTT, TT, and PT appropriately and ameliorated the blood hypercoagulability of OVX rats, which might be associated with reduced plasma vWF levels and inhibition of platelet adhesion and aggregation. Hemorheology revealed that the whole blood, plasma, and cassone viscosities of OVX rats increased, and HGWD reduced their plasma and cassone viscosities. A previous study found that HGWD can enhance blood rheology indicators (8), which is consistent with our study findings, indicating that HGWD can improve the blood flow state of microcirculation.

Furthermore, inflammation significantly contributes to myocardial microvessel disorders. Estrogen deficiency-induced excessive inflammation is a contributing factor to CVDs in postmenopausal women (21). In this study, there was an observed elevation in the serum levels of IL-1β and IL-6. These inflammatory response-mediated leukocyte adhesion and cascade reactions may result in endothelial cell dysfunction (22), exacerbating myocardial microcirculation disorders. A previous study reported that HGWD can inhibit the TNF-α/IL-6 inflammatory pathway (23). Our study demonstrated that HGWD decreased serum levels of IL-1β and IL-6 to protect microvascular endothelial function.

The *Firmicutes* to *Bacteroidetes* ratio (F/B) is generally used to assess the status of metabolic diseases. Previous studies reported that the imbalance between the F/B ratio is predominantly associated with energy metabolism (24, 25), subsequently leading to the development and progression of multiple CVDs (26). This study demonstrated a relative increase in *Firmicutes* and a relative decrease in *Bacteroidetes* in the OVX group, while HGWD treatment exhibited a mild increasing trend in the F/B ratio. The phylum of *Actinobacteria* was significantly increased in the HGWD and E groups compared to the OVX group in response to HGWD and E treatment. *Actinobacteria* break down different food components, including carbohydrates, proteins, lipids, and glycan, into a usable form, thereby ensuring a continuous caloric supply to the body (27).

At the genus level, the abundance of *Prevotella_9*, *Alloprevotella*, and *Parasutterella* in the OVX group was comparatively decreased, while *Ruminococcus_1*, *Ruminococcaceae_UCG-014*, and *Roseburia* were relatively increased compared to the SHAM group. *Prevotella 9* was associated with changes in cardiac structure and function, indicating a possible involvement in heart failure development (28). *Parasutterella* might be implicated in a new dietary carbohydrate-microbiome-host metabolic axis (29). The decrease of *Prevotella_9* and *Parasutterella* facilitates the development and progression of CVDs in the OVX rats. *Alistipes* is a disease-related gut bacterium that can cause inflammation (30). Our study observed that *Alistipes* decreased significantly in HGWD rats compared to OVX rats. These suggest that HGWD might ameliorate myocardial damage through gut colonization, though this requires experimental verification. *Ruminococcus_1* and *Roseburia* are anaerobic bacteria known for their production of short-chain fatty acids (SCFAs). These SCFAs play a crucial role in improving cardiac function and sustaining cardiovascular homeostasis, primarily through their anti-inflammatory and metabolic regulation (31). Elevated systemic concentrations of propionate and butyrate are toxic and can adversely affect patients with CVDs (32). Emerging evidence underscores the cardiometabolic relevance of the observed gut microbiota alterations. Ruminococcus_1 exhibits a negative correlation with circulating lipid levels (33) and is especially significant in distinguishing between CVD and non-CVD individuals (34). This genus may influence lipid homeostasis through bile acid metabolism modulation (35), though further mechanistic studies are warranted. Roseburia, a butyrate-producing commensal, attenuates systemic inflammation through Treg/Th17 cell balance modulation and enhanced intestinal barrier integrity (36, 37). The decrease in Roseburia abundance is associated with impaired butyrate production, which may exacerbate endothelial dysfunction through the histone deacetylase pathway (38). Our study data imply that the rise in butyrate-producing bacteria following estrogen deficiency could lead to an overproduction of SCFAs in the intestine. This observation underscores the necessity for additional mechanistic investigations to elucidate the direct involvement of gut microbiota in CVDs associated with estrogen deficiency.

PICRUSt functional prediction analysis revealed that after HGWD intervention, carbohydrate digestion and absorption, lipid metabolism, fatty acid metabolism, and lipopolysaccharide biosynthesis were enhanced, indicating that HGWD may regulate glucose and lipid metabolism *in vivo* by improving the structure and function of intestinal flora in OVX rats. A previous study reported that gut microbiota can regulate fat storage and host metabolism, which is essential for examining lipid and energy metabolism in the body (39). Moreover, estrogens are fundamental in controlling energy balance and glucose homeostasis (40). The disorder of lipid metabolism in the body is closely related to CVDs, and the accumulation of lipids can cause CVDs (41). While HGWD appears to modulate lipid metabolism through intestinal flora, potentially mitigating postmenopausal CVDs, additional metagenomic and experimental evidence is required to confirm this mechanism.

OVX rats are commonly used to study postmenopausal hormonal changes and related diseases, as they can partially mimic the physiological and pathological alterations seen in human menopause. However, several differences exist. First, human menopause involves a gradual decline in estrogen levels, whereas the OVX model induces an abrupt estrogen depletion via surgery. This may fail to fully replicate the long-term metabolic adaptations seen in natural menopause. Second, while OVX rats recapitulate postmenopausal dyslipidemia and endothelial dysfunction, differences exist in atherosclerosis progression rates and plaque composition compared to humans. These discrepancies may arise from species-specific cholesterol metabolism and vascular remodeling mechanisms. Third, given the shorter lifespan of rodents, this study focuses on early-stage cardiovascular impairments. In contrast, postmenopausal cardiovascular events in humans often result from prolonged metabolic dysregulation.

Endothelial dysfunction is one of the primary underlying mechanisms of microvascular damage, with eNOS serving as a key regulatory factor. In this study, we measured the level of VEGF and the ratio of eNOS/ET-1 in plasma. We focused on changes in the gut microbiota and cardiac microvascular endothelial function following ovariectomy. Future studies should investigate the molecular mechanisms by which HGWD may involve endothelial function in OVX rats through the classic VEGF/PI3K/Akt/eNOS pathway.

### Conclusion

HGWD exerts cardioprotective effects in OVX rats, potentially through myocardial microvascular remodeling and modulation of gut microbiota composition. HGWD is a promising therapeutic candidate for estrogen deficiency-related cardiovascular dysfunction.

## Data Availability

Data will be made available on request.
